# Assessing a Sensory-Motor-Cognition Triad in Amnestic Mild Cognitive Impairment With Dichotic Listening While Walking: A Dual-Task Paradigm

**DOI:** 10.3389/fnagi.2021.718900

**Published:** 2021-11-12

**Authors:** Marta Maria Gorecka, Olena Vasylenko, Knut Waterloo, Claudia Rodríguez-Aranda

**Affiliations:** ^1^Department of Psychology, Faculty of Health Sciences, UIT The Arctic University of Norway, Tromsø, Norway; ^2^Department of Neurology, University Hospital of North Norway, Tromsø, Norway

**Keywords:** dual-task, amnestic mild cognitive impairment (aMCI), hearing loss, gait, aging, dichotic listening

## Abstract

A contemporary topic in aging research relates to the significance of cognitive changes proper to mild cognitive impairment (MCI) to higher risk of falls and gait deteriorations. The present study addresses this question in the amnestic type of MCI (aMCI) by examining a triad of interrelated comorbidities occurring in the MCI condition: attentional impairments, hearing loss and gait disturbances. To this end, we applied a dichotic listening (DL) test during over-ground walking. DL assesses spontaneous and lateralized auditory attention in three conditions (i.e., free report or Non-forced (NF), Forced-Right (FR) ear and Forced-Left (FL) ear). Earlier reports suggest that this dual-task paradigm evoke asymmetric gait effects on healthy controls, which are moderated by degree of hearing loss. Therefore, the aim of the present study was to evaluate the effects of DL on bilateral (data from both limbs) and lateralized (each limb separately) gait outcomes in a group of forty-three aMCI participants (mean = 71.19) and fifty-two healthy older controls (mean = 70.90) by using hearing loss as a covariate in all analyses. Results showed the aMCI group presented overall compromised gait parameters, especially higher gait variability in all DL conditions during lateralized attentional control. These findings were observed bilaterally, and no lateralized effects on gait were observed. Only after controlling for hearing acuity, gait asymmetries on step length variability emerged almost exclusively in healthy controls. It was concluded that hearing loss in the aMCI group together with higher attentional impairments preclude aMCI individuals to properly execute DL and therefore, they do not display gait asymmetries. The present data demonstrate that varied demands on attentional control dependent on hearing acuity affects gait negatively in healthy older adults and aMCI individuals in very different ways. The appearance of asymmetric effects seems to be a perturbation related to normal aging, while the lack of asymmetries but exaggerated gait variability characterizes aMCI. The present findings show the intricate interplay of sensory, cognitive, and motor deteriorations in different group of older adults, which stresses the need of addressing co-occurring comorbidities behind gait perturbations in individuals prone to develop a dementia state.

## Introduction

Mild Cognitive Impairment (MCI) is the transitional stage between normal aging and dementia, which is characterized by objective impairment in one or more cognitive domains, preserved activities of daily living, and absence of dementia (Petersen et al., 2001; Winblad et al., 2004). In recent years, motor dysfunctions such as gait impairments have been associated with MCI (Verghese et al., 2008; Montero-Odasso et al., 2014; Bahureksa et al., 2017; König et al., 2017). For instance, during regular walking, individuals with MCI show slower gait velocity, shorter steps and stride length, and increased gait variability (Verghese et al., 2008; Montero-Odasso et al., 2012). These gait changes are associated with progression to dementia (Verghese et al., 2008; Doi et al., 2014; Beauchet et al., 2016; Bahureksa et al., 2017; Montero-Odasso et al., 2017; Goyal et al., 2019). For this reason, understanding the underlying causes of gait deteriorations in MCI is a central topic of investigation.

Various studies based on dual-tasks protocols have demonstrated that individuals with MCI show more deteriorated gait outcomes as compared to healthy controls, as well as worsen performance in the concomitant cognitive task (e.g., Martin et al., 2013). Thus, it is proposed that dual-task assessment may help in differentiating MCI subtypes (Montero-Odasso et al., 2014; Savica et al., 2017; Ghoraani et al., 2021). Since MCI is a heterogeneous condition with a broad range of preclinical impairments, it has been categorized into various subtypes such as amnestic, non-amnestic, single, and multi-domain types (Petersen et al., 2001). Nevertheless, at present research on MCI subtypes and gait impairments is rather scarce and inconsistent. For example, some studies addressing the matter have reported that individuals with amnestic MCI show slower gait and higher gait variability than non-amnestic MCI (Verghese et al., 2008; Doi et al., 2014); but the contrary has also been reported (Allali et al., 2016).

Because of all the MCI subtypes the most prone to progress into Alzheimer’s disease is the amnestic type (aMCI) (Petersen, 2004; Winblad et al., 2004; Ward et al., 2013), investigation of gait alterations in aMCI needs to be pursued. However, in order to address the issue, implementation of a dual-task paradigm evaluating cognitive dysfunctions associated with aMCI such as memory and attentional/executive dysfunctions (Brandt et al., 2009; Johns et al., 2012) is required. Ideally, such a paradigm should resemble a daily action, that can be experimentally tested, and which evaluates various levels of cognitive loading. The need for stringent methods that are sufficiently ecologically valid for MCI individuals is central as MCI individuals show more difficulties on task prioritization (Lee and Park, 2018) and ecological relevance determines task priority (Doumas and Krampe, 2015).

Attempts to find appropriate cognitive tasks that enable the disclosure of gait alterations in aging populations have been conducted, such as the proposal by the Canadian Consortium on Neurodegeneration in Aging (CCNA, Montero-Odasso et al., 2019). This initiative suggests the use of specific tests in dual-tasking for optimization of the assessment of cognitive-motor interaction in aging populations. Most of the suggested tasks from the CCNA consortium are mental tracking tests, which have shown to be well-suited instruments challenging gait (Al-Yahya et al., 2011). In spite that robust data supports the use of these tasks in dual-task settings, there are serious limitations related to their ecological validity as well as their lack of specificity on the type of cognitive mechanisms measured (Gorecka et al., 2018). In addition, most of these tasks rely on varied sensorial modalities. For these reasons, our group has implemented a dichotic listening (DL) test, which has proven to be ecologically valid for older adults (Gorecka et al., 2018). Indeed, DL has advantageous features for its implementation on dual-task paradigms. To begin with, DL is a robust neuropsychological procedure assessing divided and sustained attention (Kimura, 1967), as well as various aspects of executive control (Hugdahl et al., 2009) in the auditory modality. Additionally, the neural mechanisms underlying DL have been largely explored (Ocklenburg et al., 2014). In DL, different auditory stimuli are presented simultaneously to both ears, and subjects are asked to ignore or report the most salient sound or focus on a single ear. Right-handed individuals display a right-ear advantage (REA) due to the decussation of dominant language-processing in the brain (see Bryden, 1988). To date, few studies have assessed DL on individuals with MCI. The limited findings have shown that individuals with MCI fail to allocate attention to the left -side and simultaneously ignore right-side information due to a failure to sustain attention and inhibit stimuli (Andersson et al., 2008; Takio et al., 2009; Bouma and Gootjes, 2011; Utoomprurkporn et al., 2020). The same difficulty also exists to a lesser extent in cognitively healthy older adults, which indicates that focusing attention to the left-side poses the heaviest load in allocation of cognitive resources among older people (Andersson et al., 2008; Takio et al., 2009; Bouma and Gootjes, 2011; Kompus et al., 2012; Passow et al., 2012; Westerhausen et al., 2015). Interestingly, most investigations using DL in MCI populations have been conducted for the evaluation of auditory function connected to the development of dementia (Idrizbegovic et al., 2011; Häggström et al., 2018; Swords et al., 2018). This piece of information linking auditory function, DL and dementia development in MCI is noteworthy for the present study.

In our laboratory, we have applied a DL paradigm with different attentional conditions during over-ground walking in healthy older adults. Our first study (Gorecka et al., 2018), showed important asymmetrical effects on spatiotemporal measures of gait that were modulated by degree of hearing loss in cognitively normal older participants. Because the incidence of central auditory dysfunction is higher in elders with MCI (Idrizbegovic et al., 2011), the application of DL during walking will allow for the evaluation of factors known to interact with gait and which are particularly affected by the MCI condition. Taken the above facts together, the present study aims to apply the same dual-task paradigm as in our previous investigations to individuals with amnestic MCI. The main goal is to determine whether an aMCI group show quantitative or qualitative impairments on gait as compared to healthy age-matched controls. According to Simoni et al. (2021) quantitative impairments in gait are related to perturbations on typical spatio-temporal parameters such as gait speed, step length or step width, while qualitative impairments concern alterations on gait harmony, that is on symmetric outcomes of gait. Relying on the literature about MCI development and gait (e.g., Montero-Odasso et al., 2014), quantitative changes are expected to arise in the aMCI group in terms of more exaggerated deteriorations across all spatiotemporal parameters. However, we also expect to obtain qualitative dysfunctions unique to the aMCI group, such as clear asymmetric gait outcomes in those conditions with high attentional load, which according to our own data (Gorecka et al., 2018; Castro-Chavira et al., 2021) would arise during the DL conditions where spontaneous attention and attention to left side are required. Since our previous studies showed a modulating effect of hearing status on gait and DL, we also expect that higher hearing difficulties in aMCI participants will moderate the effects on gait induced by the dual-task procedure.

## Materials and Methods

### Participants and Evaluations

#### MCI Group

Sixty individuals diagnosed with MCI by a senior geriatrician or neurologist at the Department of Geriatrics and the Department of Neurology at the University Hospital of North Norway (UNN), Tromsø were recruited for the study. These individuals were referred to the specialists initially for the assessment of memory problems and they were diagnosed with F06.7 Mild cognitive disorder in accordance with the International Statistical Classification of Diseases and Related Health Problems (ICD-10) criteria. All these individuals underwent detailed examinations at the hospital that included standard laboratory and cognitive tests as well as brain imaging assessments. Inclusion criteria for this group was a referral from the specialists with a MCI diagnosis, being right-handed, Norwegian native speaker, not depressed and able to move and walk freely.

#### Older Adults in the Control Group

Fifty-eight, age-matched older adults volunteered as control participants through advertisements at the local senior citizens’ center, flyers, and as well as by means of word of mouth. Inclusion criteria for this group were being right-handed and native Norwegian speakers; free from any musculoskeletal, neurological or walking difficulties and no symptoms of clinical depression or cognitive impairment. To rule out any of the above criteria, all participants completed a semi-structured interview to collect information about their health status and health history, education and daily functioning. Furthermore, all participants were screened for depression using the Beck Depression Inventory II (BDI-II; Beck et al., 1996) and for global cognitive status with the Mini-Mental State Examination (Folstein et al., 1975)—Norwegian version (MMSE-NR; Strobel and Engedal, 2008). Only participants with a of MMSE cut-off score > 27 and not depressed according to the adapted criteria on BDI for older adults (Rodríguez-Aranda, 2003) were recruited for the study.

#### General Initial Evaluation for Both MCI and Older Volunteers

Although the MCI participants were screened for depression and global cognitive status at the University Hospital, we tested them for these aspects after enrollment in the study to standardize dataset of this investigation. Thus, all participants, both MCI individuals and older controls were evaluated with the MMSE-NR (Strobel and Engedal, 2008), the Beck Depression Inventory II (BDI-II; Beck et al., 1996), and the Falls Efficacy Scale International (FES-I; Yardley et al., 2005) to check for fear of falling. In addition, the Norwegian version of the F-36 questionnaire (Loge et al., 1998) was also applied to check the participants’ subjective evaluation of their health status and the Handedness Inventory (Briggs and Nebes, 1975) to confirm hand preference.

As part of a major umbrella project at the Department of Psychology, UIT—The Arctic University of Norway, Tromsø, about motor functions and cognition in aging, this study was approved by the Regional Committee for Medical and Health Research Ethics—REK (2009/1427). Written and informed consent was acquired from all participants prior to the study.

#### Procedures and Assessments

Even though, the main goal of this investigation is to assess a dual-task paradigm with dichotic listening (DL) while walking, there are various prerequisites necessary to perform before the dual-task paradigm could be carried out. All the participants needed to be tested with a neuropsychological battery to define and assure their group affiliation (amnestic MCI, vs. controls). Also, they needed to undergo audiometric screening to settle their hearing status and assure their hearing was well enough to perform the DL task. Thereafter, the dual-task paradigm could be performed. A clear description of this paradigm involves the methods for acquisition of gait parameters, DL-testing, and conduction of the dual-task paradigm. Based on the above, in the following section we will first present the methods related to the prerequisites (neuropsychological and audiometric assessments). Next, we will present the methods related to the dual-task paradigm (i.e., recording of gait parameters, DL test, and dual-tasking). Finally, a description of the overall data acquisition will be given.

##### Neuropsychological Assessment and Group Assignment

Since the present investigation aims to evaluate amnestic MCI participants against cognitively healthy age-matched controls, a thorough neuropsychological assessment was conducted. This allowed us to assign participants referred from the hospital to a particular MCI subgroup (i.e., amnestic, non-amnestic, multiple domain) and to corroborate that older volunteers conforming the control group were indeed free of cognitive impairments. To this end, we employed eleven neuropsychological tests to assess three cognitive domains:

###### Executive Function/Working Memory/Attention Domain

For assessment of this domain, we relied on four tests. The subtest Digit Span backward from Wechsler Adults Intelligence Scale 4th Edition (WAIS-IV, Wechsler, 2008) which examines attention and working memory was used. Also, the interference part word/color of the Stroop Word Color Test (Golden, 1978) and the Trail Making Test B (TMT B; Reitan and Wolfson, 1993) were used to examine executive functions, like inhibition and cognitive flexibility. Finally, the phonemic fluency test (COWAT, Benton, 1969) was applied to assess inhibition, ability to initiate systematic lexical search and working memory.

###### Memory Domain

Logical Memory I and II from Wechsler Memory Scale 3rd edition (Wechsler, 1997); the subtest Digit Span forward from Wechsler Adults Intelligence Scale 4th Edition (WAIS-IV, Wechsler, 2008), as well as semantic fluency (Newcombe, 1969) were used to measure memory abilities.

###### Visuospatial Abilities Domain

Visuospatial processing was examined by applying Block Design from WAIS-IV (Wechsler, 2008), the Clock Drawing Test (CDT, Shulman, 2000) and Trail Making Test A (TMT A; Reitan and Wolfson, 1993). The first two are tests commonly used to evaluate visual memory and construction ability, while the TMT A is employed in the assessment of visuospatial ability, motor skills in addition to processing speed.

###### Procedures for Group Assignment

Mild cognitive impairment participants in this study were classified according to neuropsychological criteria of MCI suggested by Jak et al. (2009) and Bondi et al. (2014). These criteria propose that in order to qualify as MCI in a particular domain, it is required that an individual shows impaired performance greater than one standard deviation (SD) below appropriate age-norms. Thus, participants in the MCI group were classified as amnestic MCI if they were impaired on tests belonging to the memory domain. As part of a major aging project at our institution, we also classified the referred patients into the MCI categories of non-amnestic (if they presented impairment in a non-memory domain) and of multiple domain (if they presented impairment in various cognitive domains). As for the older volunteers, they were confirmed as cognitively normal, if their performance on each of the assessed domains was within 1 SD of the normative expectations.

#### Audiometric Screening

All participants completed audiometric screening in a double-walled, sound- attenuated room using pure-tone audiometry (Madsen Itera II, GN Otometrics, Denmark). Hearing sensitivity was measured calculating the Pure Tone Average (PTA) from hearing thresholds of the frequencies 500, 1,000, 2,000, and 4,000 Hz. The results of the PTA showing thresholds > 45 dB scores as well as an interaural difference larger than 15 dB were criteria for exclusion of participants (Saliba et al., 2009).

#### Acquisition of Gait Parameters

Spatiotemporal parameters of gait were acquired using the OptoGait System (OptoGait, Microgate, Bolzano, Italy). The system consists of transmitting-receiving bars aligned in parallel and creating a 7 × 1.3 m area that quantifies spatiotemporal gait parameters by using photoelectric cells that register interference in light signals. The sensors in the OptoGait system are placed over ground in a rectangular fashion where subjects walk within in circles. Ninety-six LED diodes are positioned on each bar one centimeter apart at three millimeters above the ground. When subjects pass between two bars positioned in parallel with the ground, transmission and reception are blocked by their feet, automatically calculating spatio-temporal parameters. Data were extracted at 1,000 Hz and saved on a PC using OptoGait Version 1.6.4.0 software. Gait parameters examined were gait speed, step length, and step width, for both feet and per foot. Linear measures including the mean (M) and the coefficient of variation [CoV, based on the formula (SD/mean) × 100%] were calculated for each gait parameter. All walking conditions were recorded with two Logitech web cameras from different angles to overlook any difficulties or changes during walking conditions. The Optogait system has proven to be a highly reliable and valid instrument (Lee et al., 2014).

#### Dichotic Listening Task

As the concomitant cognitive task to walking, we applied the Bergen Dichotic Listening Test (Hugdahl and Andersson, 1986). The test consists on the simultaneous and randomized presentation of six syllables: /ba/ /ta/ /pa/ /ga/ /da/ /ka/. Each pair of syllables has a duration of 350 ms. The syllables were paired with each other in all possible combinations to form 36 different syllable pairs. From these, the homonymic pairs (e.g., ba–ba) were included in the test as perceptual control, but not considered in statistical analysis. The syllables were read by a Norwegian-speaking male voice with constant intonation and intensity with a time interval of 4,000 ms. The total duration of each DL condition was 3 min. The DL procedure has three conditions: The Non-forced condition (NF) was always conducted first where participants were instructed to report the syllable they heard the clearest. The NF condition evaluates spontaneous attentional abilities as subjects choose freely which stimulus they report. Thereafter, two conditions followed where participants were instructed to pay attention either to the right ear (Forced-Right condition, FR) or to left ear (Forced-Left condition, FL) while ignoring stimuli from the opposite ear. The forced attention conditions evaluate volitional lateralized attentional control to the respective side. On each DL-condition, the following scores are calculated: Number of correct responses and homonyms, number of errors/no responses and calculation of a laterality index (LI = $\left[\frac{\left(RE-LE\right)}{RE+LE}\right]\times 100$) for each condition. These scores were used in the statistical analyses for DL. The FR and FL were counterbalanced across subjects depending on their ID number. Participants with ID numbers that were odd numbers received FR before FL. The syllables were presented using wireless noise-canceling headphones.

### Dual-Task Paradigm

**FIGURE 1:**
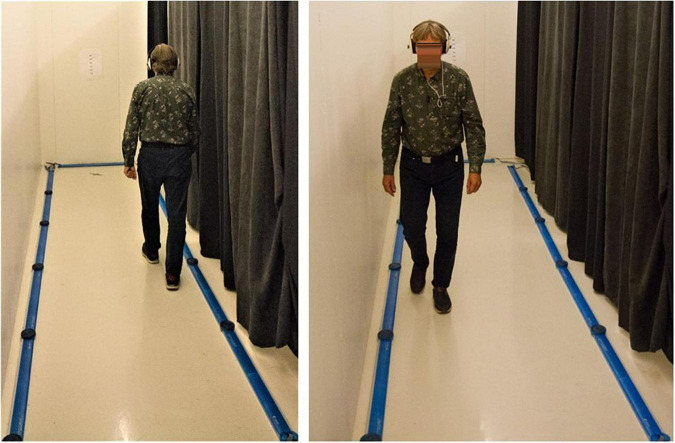
Illustration of the dual-task experimental setting and a volunteer performing the paradigm.

In this part of the study, all participants were evaluated for single walking as well as while performing the three DL conditions (i.e., Non-forced, NF; Forced-Right, FR; Forced-Left, FL) during walking. Thus, four conditions confirmed the paradigm: (1) A baseline walking condition (i.e., only walking); (2) NF while walking; (3) FR while walking; and (4) FL while walking. It is important to remark that no previous training or habituation sessions were conducted as we aimed to obtain data from naïve subjects exposed to a single experience. The experiment was conducted in a rectangular shaped room. An illustration of the experimental setting is shown on Figure 1.

#### Baseline Condition (Only Walking)

Prior to the experiment, participants were given a demonstration trial of the walking direction by the experimenter within the gait analysis system, and they were required to confirm the well understanding of these instructions. To allow for the best ecological valid situation, participants were asked to walk in a self-selected, comfortable walking speed (usual), counterclockwise. The decision of the counterclockwise direction agrees with the natural tendency of right-handed individuals to turn to the left (e.g., Mohr et al., 2003; Lenoir et al., 2006). The Optogait system started recording the gait measures when the subject took their first footstep, initiated by a verbal signal. In the baseline condition, participants were instructed to walk for 1 min within the Optogait field to collect baseline measurements without performing the cognitive task. Based on pilot trials, the baseline condition was shorter (1 min) than the rest of the dual-task conditions (3 min). The reason was to obtain a balanced situation in which subjects did not get tired or lightheaded while allowing acquisition of enough gait data.

#### Preparations for Dual-Tasking

Participants were given a demonstration trial of how to perform the DL before the dual-task was conducted. First, the experimenter explained to the subjects that they will wear headphones while walking again at their usual pace, and that they will be exposed to different syllables on each ear. Participants were also asked to wear around the neck a small portable digital recorder to record their responses during the trial. A sheet of paper with the six printed syllables used in DL test was shown to the participants to clarify the sort of stimuli used. A similar sheet of paper was attached on one wall at the end of the walkway to remind participants which stimulation they should expect. Then, they were required to listen and respond loudly to three stimuli presentations from the DL test while wearing headphones in a stand still position. In this way, we ensured good comprehension of the instructions. Volume of the auditory stimuli was also adjusted for each person prior to the testing. Moreover, we emphasize equal task prioritization by asking subjects to keep walking and execute the DL task as accurate as possible during the entire trial.

#### DL Instructions

*DL Non-forced condition:* In this condition participants were asked to report loudly the syllables best perceived. Instructions were: “We ask you to loudly say the clearest syllable you detect each time you get stimulation. Please walk at your usual pace all the time while responding. We remind you that only six possible syllables (those shown on the paper) will be presented and we ask you to perform as well as possible walking continuously in rounds in the designated area as previously demonstrated, while reporting the clearest sounds you perceive.” *DL Forced-Right condition:* In this condition, same instructions were given with the only difference that we required subjects to report loudly only the syllables presented to the *right ear*. *DL Forced-Left condition:* Again, same instructions yielded, but this time participants were asked to report syllables presented to the *left ear*.

#### Conduction of DL While Walking

In all dual-task conditions, the dichotic listening task was initiated simultaneously as the subject lifted a foot to initiate walking, again when the experimenter gave a verbal cue. At the time of testing, DL-responses were recorded in the digital voice recorder and also written down by the experimenter on a sheet of paper. Data acquisition for gait parameters were conducted in the 1-min trial for baseline and on each 3-min trial of the DL conditions during walking. When necessary, short breaks were given between baseline and on each of the dual-task conditions. After the experiment was completed, two additional experimenters listened the recorded DL answers from the digital recorder and checked them against the written answers to ensure the reliability of the data. Thereafter, the experimenters manually inserted all DL answers in the E-prime 2.0 Software (Psychology Software Tools, Inc., Pittsburgh, PA, United States) for the calculation of DL scores.

#### General Procedure for All Data Acquisition

The study took place at the Department of Psychology, UiT Arctic University of Norway. The duration of the whole procedure was about 3 h and testing sessions were divided into two sessions to avoid fatigue. In the first session, participants were interviewed to acquire their demographic background and health history in a sound-attenuated room. Also, in this session and under the same environment, they underwent audiometric screening, and they were evaluated with the neuropsychological test battery. In the second session, participants answered to remaining questionnaires and they performed the dual-task paradigm.

### Statistical Analyses

All analyses were performed with the statistical package IBM SPSS Statistics 26. Group comparisons for demographics, background variables, cognitive tests and questionnaires were performed with independent *t*-tests.

#### Classification of MCI Subgroups

We applied the method used by Aarsland et al. (2009), where raw cognitive scores were converted into z-scores using the mean and standard deviations of an existing database of cognitively healthy older adults (*n* = 103) from North Norway collected at our laboratory. Thereafter, an averaged composite score by domain was calculated for each participant. Adjustments regarding age, sex and education were performed *via* multiple regression analyses relying on the cognitively healthy older adults’ database for each cognitive domain. The intercepts and beta weights from these calculations were used to obtain predicted z-scores for each participant in the study.

#### Dichotic Listening Data

A series of factorial analyses of variance with repeated measures in one factor were carried out. For DL data, the design 2 Group (aMCI, Control) × 2 Ear (right, left) × 3 Condition (NF, FR, and FL) was used. In case of a significant omnibus test, univariate tests were performed. Multivariate tests for simple main effects were employed in the case of significant interactions.

#### Gait Parameters

For gait, we also applied a series of factorial analyses of variance with repeated measures in one factor. This time, we analyzed the mean and coefficient of variations (CoV) separately for each gait parameter. First, we analyzed bilateral outcomes (i.e., data from both limbs together) and then lateralized outcomes (i.e., separate data for each limb). In the first set of analyses, mixed-ANOVAs were conducted for bilateral gait parameters with the design 4 Condition (Baseline, NF, FR, and FL) × 2 Group (aMCI, Control). Next, we investigated the existence of possible asymmetric effects on gait parameters due to the DL condition with the mixed-ANOVA design of 4 Condition × 2 Group × 2 Feet. In case of a significant omnibus test, univariate tests were performed. Multivariate tests for simple main effects were employed in the case of significant interactions.

#### Effects of Hearing Loss on DL and Gait

Since age-related hearing impairment has shown to modulate effects of lateralized attention on gait parameters in previous studies from our laboratory (Gorecka et al., 2018), we performed different ANCOVAs by using Best PTA as covariate. In this investigation, the moderating effects of hearing status were explored on both gait and on DL data. The use of Best PTA was chosen as it depicts the lowest functional threshold, which enables hearing compensation (Linssen et al., 2014).

In all analyses, Greenhouse–Geisser corrections were chosen when the sphericity assumption was not met. Significant interactions or main effects involving group differences were followed up with appropriate *post hoc* analyses. The Bonferroni correction was applied across all factorial analyses.

## Results

### Group Assignment

By applying a cut-off of ≥ 1 SD lower than the expected z-score on the memory domain we were able to identify 43 amnestic MCI individuals from the original pool of 60 referred participants. As for the control group, we were able to confirm that 52 out of 58 older adults recruited originally as control volunteers were cognitively healthy and thus, these participants were retained for the present study.

### Demographics

Results from demographic variables are shown in Table 1. No significant differences were found between the groups regarding age or education. Positive measures from the Handedness Inventory confirmed participants were right-handed. However, significant group differences were found where the aMCI group reported significantly more preference to the use of right hand than healthy controls. No group differences were found in terms of self-reported health status, fear of falling or depression.

**TABLE 1:** Demographic characteristics and initial assessments by group.

	Controls (*n* = 52)	aMCI (*n* = 43)	
		
Gender (men/women)	24/28	20/23	
	M (SD)	M (SD)	*t*
Age	70.90 (7.35)	71.19 (8.75)	0.17
Education (years)	13.18 (3.55)	11.90 (3.91)	–0.65
MMSE-NR	29.27 (1.07)	25.67 (3.28)	−7.45***
BDI-II	5.39 (5.46)	6.08 (4.77)	0.21
Handedness	19.88 (4.47)	22.02 (3.37)	2.56*
FES–I	19.26 (4.02)	19.66 (4.67)	0.41
SF-36	111.98 (45.52)	105.00 (9.62)	0.31
PTA right (dB)	23.74 (11.21)	32.69 (16.17)	3.17**
PTA left (dB)	25.56 (11.83)	32.09 (13.88)	2.47*
PTA Worst (dB)	27.45 (12.53)	35.41 (16.90)	2.68**
PTA Best (dB)	21.53 (9.62)	29.37 (12.90)	3.39***

M, mean; SD, standard deviation; MMSE-NR, mini mental status examination - norwegian revision; BDI-II, Becks Depression Inventory; FES-I, Falls Efficacy Scale International; SF-36, Short Form Survey 36 items; PTA, Pure Tone Average; dB, decibel. *p ≤ 0.05; **p ≤ 0.01; and ***p ≤ 0.001.

### Audiometric Scores

Table 1 also shows pure tone average scores interaurally for both groups. The aMCI group had significantly higher hearing thresholds compared to healthy controls on all outcomes.

### Neuropsychological Results

Results from the neuropsychological assessments are displayed in Table 2. The control group showed significantly better performances than aMCI individuals on all neuropsychological measures.

**TABLE 2:** Means and standard deviations from neuropsychological tests by cognitive domain.

	Controls (*n* = 52)		aMCI (*n* = 43)		
Domain	M	(SD)	M	(SD)	*t*
**Executive functions/working memory**					
TMT B, sec	92.10	(27.56)	195.67	(157.53)	5.04***
Stroop WCI	32.52	(8.87)	27.63	(11.61)	−2.24*
DigitSpan B	8.08	(1.49)	6.37	(1.59)	−5.32***
COWAT	13.83	(3.21)	11.16	(4.17)	−3.13**
**Memory**					
Log Memory I	23.93	(6.04)	8.16	(6.13)	−10.14***
Log Memory II	27.56	(6.09)	7.39	(8.17)	−11.74***
DigitSpan F	8.90	(1.76)	7.37	(1.28)	−4.69***
Sematic Fluency	16.54	(3.52)	12.78	(4.27)	−4.13***
**Visuospatial**					
CDT	6.93	(0.23)	6.10	(1.71)	−3.49**
TMT A, sec	36.07	(15.09)	56.64	(24.81)	5.31***
Block Design	38.23	(8.02)	27.61	(9.76)	−5.76***

M, Mean; SD, standard deviation; aMCI, amnestic Mild Cognitive Impairment; CDT, Clock Drawing Test; TMT, Trail Making Test; Sec, seconds; Stroop WCI, Stroop Word-Color Interference; COWAT, Controlled Oral Word Association Test; DigitSpan B, Digit span backward; DigitSpan F, Digit span forward; Log Memory, Logical Memory. *p < 0.05; **p < 0.01; and ***p < 0.001.

#### Dichotic Listening Results

##### Correct Responses

**FIGURE 2:**
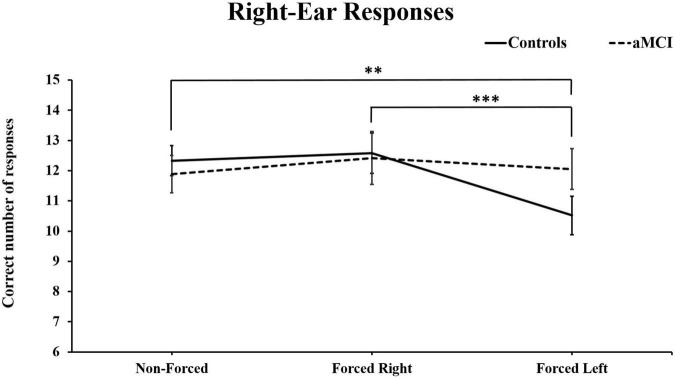
Mean and ± SEM for correct right-ear responses across three dichotic listening conditions. aMCI, amnestic Mild Cognitive Impairment. (**p < 0.01; and ***p < 0.001) Reported significant differences are only for the control group.

**FIGURE 3:**
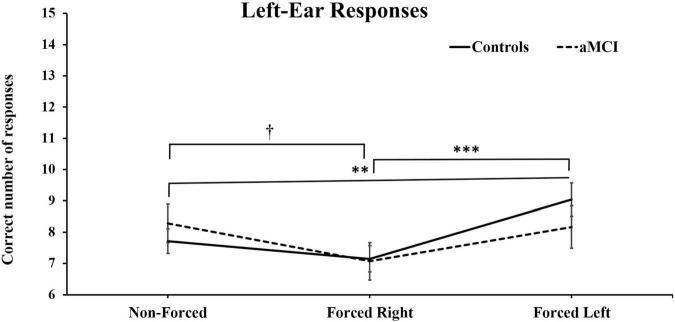
Mean and ± SEM for correct left-ear responses across three dichotic listening conditions. aMCI, amnestic Mild Cognitive Impairment. The reported † denotes significant differences of *p* < 0.05 for aMCI group and the ^∗∗^ and ^∗∗∗^ denotes significant differences of *p* < 0.01 and *p* < 0.001) respectively, for healthy controls.

Three-way MANOVA showed only significant main effect for Ear [Pillai’s Trace = 0.33, *F* (1, 93) = 46.66, *p* < 0.001, η^2^_p_ = 0.33]. No main effect of group [*F* (1, 93) = 0.040, *p* = NS] or condition were found, [Pillai’s Trace = 0.01, *F* (2, 92) = 0.045, *p* = NS] However, there was a significant interaction for Condition × Ear [Pillai’s Trace = 0.16, *p* < 0.001, *F* (2, 92) = 9.98, *p* < 0.001, η^2^_p_ = 0.16]. These results are as expected and naturally due to the change in focus of attention driven by the instructions. Additionally, an interaction effect between Condition × Ear × Group [Pillai’s Trace = 0.07, *F* (2, 92) = 3.68, *p* < 0.05, η^2^_p_ = 0.07] was observed. Simple main effects analyses of this interaction revealed that healthy controls produced significantly less correct right-ear responses in the FL conditions, as compared to right-ear responses in the NF, *p* < 0.01, and the FR conditions, *p* < 0.001 (see Figure 2). Furthermore, the cognitively healthy controls also reported significantly less from the left-ear in NF (*p* < 0.01) and FR (*p* < 0.001) compared to the FL condition. Concerning the aMCI group, these subjects reported significantly more correct left-ear responses in NF than in FR. No further significant differences were seen (see Figure 3). Controlling for effects of hearing on correct responses: After controlling for hearing, a significant interaction between Condition × Ear, [Pillai’s Trace = 0.08, *F* (2, 91) = 3.85, *p* < 0.05, η^2^_p_ = 0.08] was still present and showed same results as previously. The interaction effect was seen in healthy controls, [Pillai’s Trace = 0.13, *F* (2, 49) = 3.57, *p* < 0.05, η^2^_p_ = 0.13], but not in aMCI. However, the original significant interaction effect between Condition × Ear × Group was no longer significant [Pillai’s Trace = 0.05, *F* (2, 91) = 2.49, *p* = NS].

##### Laterality Index

Further analysis for laterality index (LI), showed a main effect of Condition, [Pillais’s Trace = 0.11, *F* (2, 92) = 5.51 *p* < 0.006, η^2^_p_ = 0.11] and an interaction effect Condition × Group [Pillai’s Trace = 0.07, *F* (2, 92) = 3.27, *p* < 0.05, η^2^_p_ = 0.16]. This interaction effect showed that the cognitively healthy control group had significantly higher REA, i.e., laterality index in the NF, 22.7% (SD = 23.43), and FR, 26.7%, (SD = 29.72) condition compared to FL, 8.4% (SD = 32.67). There were no significant differences in laterality index between the conditions in the aMCI group, with 17.7% (SD = 32.23) in the NF condition, and 24.9% (SD = 37.2) and 19.5% (SD = 36.0) in FR and FL respectively. Controlling for effects of hearing on LI: The significant interaction on LI was no longer significant when controlling for Best PTA [Pillai’s Trace = 0.04, *F* (2, 91) = 1.95, *p* = NS].

##### DL Errors and Non-responses

We distinguished the errors into real errors (commissions) and non-responses (omissions). For the errors, there was only a main effect of Condition [Pillai’s Trace = 0.15, *F* (2, 91) = 8.25, *p* < 0.001, η^2^_p_ = 0.15]. *Controlling for effects of hearing on errors/non-responses:* By controlling for hearing acuity, this effect was no longer significant. For non-responses, we also found a significant main effect of Condition [Pillai’s Trace = 0.20, *F* (2, 92) = 11.67, *p* < 0.001, η^2^_p_ = 0.20] that persisted after controlling for Best PTA [Pillai’s trace = 0.10, *F* (2, 91) = 5. 03, *p* < 0.01, η^2^_p_ = 0.10].

#### Results for Gait Outcomes

##### Bilateral Results (i.e., Right and Left-Foot Data Together)

The analyses performed with series of two-way MANOVAs on the mean and CoV values of step length [mean: Pillai’s Trace = 0.58, *F* (3, 91) = 42.72, *p* < 0.001, η^2^_p_ = 0.58; CoV: Pillai’s Trace = 0.15, *F* (3, 91) = 5.62, *p* < 0.001, η^2^_p_ = 0.16], and gait speed [mean: Pillai’s Trace = 0.57, *F* (3, 9) = 40.68, *p* < 0.001, η^2^_p_ = 0.57; CoV: Pillai’s Trace = 0.10, *F* (3, 91) = 3.69, *p* < 0.05, η^2^_p_ = 0.11] showed a main effect of condition in which shorter steps, slower speed and increased variability were found during the dual-task conditions as compared to baseline. In contrast, no main effect of condition was found for the mean [Pillai’s Trace = 0.06, *F* (3, 91) = 2.08, *p* = NS] or CoV [Pillai’s Trace = 0.06, *F* (3, 91) = 1.9, *p* = NS] of step width. No significant interactions were found.

Furthermore, a main effect of group was found in these three spatio-temporal parameters for mean values [step length: *F* (1, 93) = 21.44, *p* < 0.001, η^2^_p_ = 0.19; gait speed: *F* (1, 93) = 40.68, *p* < 0.001, η^2^_p_ = 0.20; step width: *F* (1, 93) = 26.63, *p* < 0.001, η^2^_p_ = 0.22] showing more deteriorated results in the aMCI group as compared to healthy controls. Likewise, an effect of group was found for CoVs for step length [*F* (1, 93) = 26.58, *p* < 0.001, η^2^_p_ = 0.22] and gait speed [*F* (1, 93) = 22.11, *p* < 0.001, η^2^_p_ = 0.19] where aMCI demonstrated higher variability than controls. No main effect of group was observed for CoV in step width [*F* (1, 93) = 0.14, *p* = NS] and no significant interactions were found. *Controlling for effects of hearing on bilateral gait data:* By controlling for hearing status none of the results mentioned above were modified, except for CoV of gait speed, where the main effect of condition was no longer present [Pillai’s Trace = 0.03, *F* (3, 90) = 0.92, *p* = NS]. Results from the bilateral analyses are presented in Supplementary Table 1.

##### Lateralized Results (i.e., Right Foot and Left Foot Separately)

A series of three-way MANOVAS were performed in this part of the analyses where the factors of condition (x 4), group (x 2) and foot (x 2) were tested. By conducting these lateralized analyses, we assessed possible asymmetric effects of DL on each of the gait measures. Results did not show any significant main effect of foot for neither gait speed [mean: Pillai’s Trace = 0.01, *F* (1, 93) = 1.29, *p* = NS; CoV: Pillai’s Trace = 0.01, *F* (1, 93) = 0.73, *p* = NS] or step length [mean: Pillai’s Trace = 0.01, *F* (1, 93) = 1.13, *p* = NS; CoV: Pillai’s Trace = 0.0, *F* (1, 93) = 0.16, *p* = NS]. The same results as those reported in the bilateral analyses regarding main effects for condition and group were replicated for these two variables on both means and CoVs values and therefore, these results are not reported in this section.

However, a main effect of foot was observed on the mean of step width [Pillai’s Trace = 0.11, *F* (1, 93) = 11.30, *p* < 0.001, η^2^_p_ = 0.11]. This finding indicated that the values of step width were wider for the right foot of all participants disregarding group. Furthermore, the main effect of group already observed in the bilateral analyses was equally present in the lateralized analyses. Nevertheless, this time we could note that the differences between feet were larger for aMCI group than for controls (see Table 3). In spite of this finding, no significant Foot × Group interaction existed. Interestingly, no main effect of foot was observed on the CoV of step width [Pillai’s Trace = 0.00, *F* (1, 93) = 0.41, *p* = NS], which agrees with the lack of main effects for group and condition already observed on this exact variable in the bilateral MANOVA.

**TABLE 3:** Mean and standard deviations for gait parameters by foot expressed in mean values and coefficients of variation (CoV).

	Conditions									
	Baseline		Non-forced		Forced-right		Forced-left			
	Controls	aMCI	Controls	aMCI	Controls	aMCI	Controls	aMCI	RMANOVA, *p*, (η^2^_p_)	ANCOVA, *p*, (η^2^_p_)
	M (SD)	M (SD)	M (SD)	M (SD)	M (SD)	M (SD)	M (SD)	M (SD)	Condition/Foot/Interact./Group	Foot/Interact./Group/PTA
**Mean**										
Step length R	62.8 (12.4)	55.4 (8.8)	60.4 (10.1)	51.6 (9.4)	58.7 (10.0)	50.4 (8.3)	58.9 (9.9)	50.5 (8.9)		
Step length L	63.9 (9.0)	55.5 (8.9)	60.7 (9.2)	51.9 (9.2)	59.2 (9.4)	50.3 (9.4)	59.2 (9.4)	50.3 (9.4)	0.001 (0.3)/ NS/ NS//0.001 (0.2)	NS/0.026 (0.1)/0.003 (0.1)/0.003 (0.9)
Gait speed R	1.1 (0.2)	0.8 (0.2)	1.0 (0.2)	0.7 (0.2)	0.9 (0.2)	0.7 (0.2)	1.0 (0.3)	0.7 (0.2)		
Gait speed L	1.1 (0.2)	0.9 (0.2)	1.0 (0.2)	0.7 (0.2)	0.9 (0.3)	0.7 (0.2)	1.0 (0.3)	0.7 (0.2)	0.001 (0.4)/ NS /NS /0.001 (0.2)	0.03 (0.5)/NS/0.001 (0.1)/0.001 (0.3)
Step width R	9.3 (3.6)	13.5 (3.7)	9.7 (2.7)	13.2 (3.3)	10.3 (4.2)	13.1 (3.5)	9.0 (4.0)	13.2 (3.8)		
Step width L	9.0 (3.8)	12.8 (3.1)	9.3 (3.2)	13.1 (3.5)	9.7 (4.2)	13.1 (4.0)	10.6 (3.5)	13.5 (3.7)	NS / 0.002 (0.1)/ NS/ 0.001 (0.2)	NS / NS/0.001 (0.1)/0.001 (0.1)
**CoV (%)**										
Step length R	8.6 (6.2)	15.4 (7.5)	6.9 (5.7)	13.9 (7.3)	7.9 (6.5)	16.6 (9.9)	9.4 (7.9)	15.5 (7.9)		
Step length L	8.6 (7.0)	15.4 (7.1)	7.7 (7.0)	13.5 (7.4)	7.9 (5.8)	17.0 (10.0)	9.4 (7.7)	15.1 (7.5)	0.001 (0.07)/0.015 (0.04)/NS/0.001 (0.2)	0.009 (0.04)/0.001 (0.2)/0.001 (0.1)
Gait speed R	8.7 (9.8)	20.9 (11.8)	9.2 (9.0)	21.6 (10.6)	12.4 (16.1)	26.5 (35.7)	11.9 (15.1)	27.2 (42.9)		
Gait speed L	8.4 (9.1)	20.6 (11.6)	9.4 (8.6)	21.3 (10.6)	12.9 (15.2)	22.5 (35.8)	11.9 (14.7)	22.2 (11.6)	0.029 (0.03)/ NS/ NS/0.001 (0.06)	NS/NS/ 0.001 (0.13)/0.011 (0.07)
Step width R	83.2 (43.6)	90.6 (31.9)	77.1 (29.4)	85.8 (33.9)	85.5 (29.9)	85.8 (29.8)	85.7 (35.4)	86.6 (32.7)		
Step width L	81.3 (31.4)	90.6 (37.1)	82.9 (35.5)	83.0 (26.5)	87.6 (31.8)	85.5 (29.8)	86.6 (32.7)	83.6 (28.3)	NS / NS / NS / NS	NS / NS / NS/ NS

*RMANOVA and ANCOVA with Bonferroni correction for multiple comparisons. Interactions marked with †refer to = Condition × Foot *p <* 0.05. Units for Step length, Step width and Stride length = cm.; units for Gait speed = m/s. M, mean; SD, standard deviation; R, Right; L, Left; RMANOVA, repeated measures analysis of variance; ANCOVA, Analysis of covariance; CoV, Coefficient of Variation; Interact., Interactions; PTA, Best Pure Tone Audiometry values; NS, non-significant; CoV, calculated with the formula: (SD/mean) × 100.*

**FIGURE 4:**
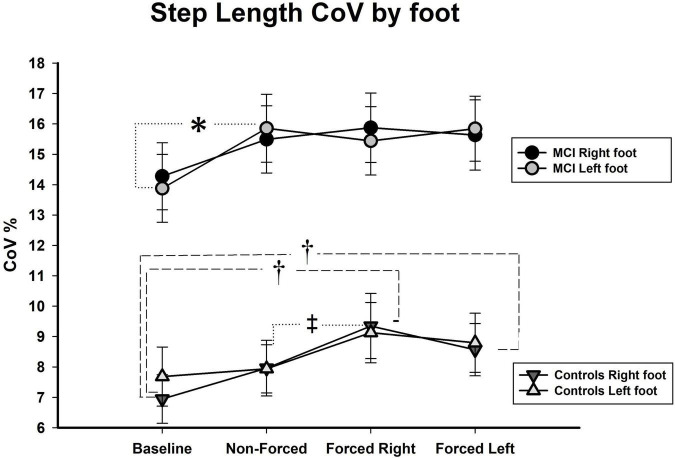
CoV, coefficient of variance, shown in %; aMCI, amnestic mild cognitive impairment; R, right; L, left; (significance level in aMCI shown with ^∗^*p* < 0.05; ^∗∗^*p* < 0.01; and ^∗∗∗^*p* < 0.001. Significance in healthy controls shown as ^†^*p* < 0.05 for one limb and ^‡^*p* < 0.05 for both limbs).

Controlling for effects of hearing on lateralized gait parameters: In line with the approach applied on the bilateral analyses, we also conducted a series of factorial MANCOVAs with Best PTA as covariate on the lateralized assessments. Results showed no significant effects of the covariate in the mean of all three gait measures or on the CoVs of gait speed and step width. Nonetheless, we found an exception for the CoV of step length in which Best PTA affected the original results by nullifying a significant interaction and causing the occurrence of two new significant interactions. First, we found that the interaction between Condition × Group became non-significant after controlling for hearing status [Pillai’s Trace = 0.08, *F* (3, 90) = 2.51, *p* = NS]. Second, the appearance of a significant interaction between Condition × Foot [Pillai’s Trace = 0.08, *F* (3, 90) = 2.75, *p* < 0.05, η^2^_*p*_ = 0.11] and between Condition × Foot × Group [Pillai’s Trace = 0.08, *F* (3, 90) = 2.63, *p* < 0.05, η^2^_*p*_ = 0.08] were seen after controlling for Best PTA. Analyses of simple main effects revealed that aMCI participants displayed significantly higher CoVs (*p* < 0.05) for left foot in the NF condition as compared to Baseline (see Figure 4). No further significant differences for the aMCI group were seen in left or right foot variability across the dual-task conditions.

Regarding the healthy control group, several findings yielded. To begin with, there was observed a significant increment in variability on both the right and the left foot (*p* < 0.05) during the FR as compared to NF condition (see Figure 4). Also, step length variability of right foot increased significantly during the FR (*p* < 0.001) and the FL (*p* < 0.01) conditions as compared to Baseline.

## Discussion

In the present study, individuals with amnestic MCI and cognitively healthy older controls performed a dual-task paradigm consisting of a dichotic listening task simultaneously to over-ground walking. The main goal was to determine whether spontaneous vs. volitional focus of attention evoked quantitative and qualitative impairments on gait in aMCI individuals as compared to healthy controls. As in any complex dual-task situation, we found that performing DL while walking compromised quantitatively all gait parameters in the aMCI group. The aMCI group showed worse mean values in all conditions, in regard to slower gait speed, shorter step length, and wider step width. However, the aMCI group’s CoVs were significantly higher for step length and gait speed during the forced attention-conditions. No increment was found on step width CoV. Thus, these data confirm that our dual-task paradigm posed heavier demands for the individuals with aMCI particularly during volitional control of attention.

However, we also expected qualitative differences in the aMCI group, such as asymmetric gait outcomes related to lateralized focus of attention. This was not the case. Only after adjusting for hearing status, we observed a significant asymmetric increment on step length variability of left foot in the aMCI group during the NF condition. No further significant asymmetries were seen in this group. It could be argued that this is a main finding in our study, but a closer scrutiny to Figure 4 shows that the result could be incidental. Indeed, the significant result offers a hint to the possible moderating role hearing loss might exert on step length variability of these individuals. Notwithstanding, we rather believe that the main finding of this investigation relies on the *lack* of asymmetries in the aMCI group. In fact, the difference in number of significant asymmetries that arose in healthy controls and not in aMCI participants after controlling for hearing status is worth noting.

After adjusting for Best PTA, significant asymmetries were disclosed in the control group related to increased step length variability of their right foot in all conditions, though the effect was more evident during the forced-attention conditions. Usually, asymmetries are regarded as deleterious or linked to pathology in older populations (Verghese et al., 2008; Yogev-Seligmann et al., 2008). Therefore, our interpretation has been that asymmetries in healthy older adults evoked by directing attention to one specific ear are detrimental. However, in the present study, without accounting for hearing status, these effects get masked and their emergence after controlling for Best PTA -thresholds suggests a link to good auditory compensation. This interpretation was also reported in our study from 2018 (Gorecka et al., 2018). In short, healthy elders have better hearing acuity than aMCIs across all audiometric outcomes, which indicates that controls had better perceptual abilities that enable them to perform the task appropriately and for this reason the asymmetries evoked may represent a risk of falling in healthy seniors.

Now, the question is why aMCIs did not display as many asymmetries as controls and the answer may rely on performance of the DL task. As mentioned in the introduction, dichotic listening tasks have been usually applied in MCI persons for the assessment of hearing ability (Swords et al., 2018), which limits information about the exact nature of attentional/executive disabilities of this population during performance of DL. In the present study, even if the aMCI group reported more responses from the right side (regardless of the task’s instructions), the number of correct responses across conditions did not differ significantly between groups. In spite of no group differences, aMCIs clearly showed a difficulty to direct attention to left ear as denoted by the laterality indexes (LIs). This finding is understandable as aMCI individuals present not only memory difficulties but also executive impairments (Johns et al., 2012; Rabi et al., 2020), which are documented by the neuropsychological results of our study. Notwithstanding, the LIs not only point to the aMCI group’s difficulty in focusing on the non-dominant ear. On one hand, LIs revealed that the aMCI group had less REA in the NF condition than healthy controls, indicating troubles in bottom-up processing based on perceptual salience of the stimulus material (Kaya and Elhilali, 2014), which can be related to their hearing difficulties. On the other hand, aMCI participants did not show a clear attentional focus for any side, as their LIs were rather similar during the NF and FR conditions. Thus, these findings suggest a lack of lateralized attentional capacity to attend auditory stimuli, which hinders them to properly direct their attention to any specific side. Still, the aMCI group’s gait becomes compromised, though, not asymmetrically.

The reason for having difficulties in the DL task during walking are various and not necessarily only based on executive dysfunctions. It is certain that the main sources of their inability to perform DL are associated to executive impairments and hearing troubles. Still, the aMCI individuals could have neglected to adequately execute the dual-task due to prioritization of walking. It has been shown that just “walking while talking” is a demanding task for some type of elders (Lundin-Olsson et al., 1997). Thus, a too complex cognitive task such as DL while walking, imposing too much cognitive load causes older individuals with cognitive impairment to take a cautious and more secure walking strategy (Cederwall et al., 2014; Montero-Odasso et al., 2014). Hence, it is possible that the aMCI group adopted a “posture first”- strategy (i.e., they might have prioritized the walking) (Yogev-Seligmann et al., 2012), in spite of being required to perform as well as possible, both the walking and attending the DL test. This highlights the importance of selecting appropriately difficulty level for the concomitant task (see Montero-Odasso et al., 2014; Bishnoi and Hernandez, 2020). For instance, the Bergen Dichotic Listening Test that was applied in this study is based on syllables as auditory stimuli. It may have proved difficult for aMCI participants to perceive and differentiate between these sounds, due to their heavier hearing difficulties. It is reported that effortful listening in older people increases cognitive load (Carr et al., 2020). Thus, not being able to fully perceive sounds successfully, might have increased the cognitive load during execution of DL and under such circumstances, auditory and motor processes may compete for limited resources (Bruce et al., 2017). Based on the above, it is plausible that implementation of another DL paradigm relying on the use of meaningful words may promote asymmetric effects as those seen in healthy controls. Therefore, future studies evaluating individuals with MCI should apply DL paradigms involving regular or familiar words for easier recognition (Westerhausen and Samuelsen, 2020) and confirmation of the present findings such as simple numbers (Klichowski and Kroliczak, 2017) or sentences like the “Dichotic sentence identification test” (Jerger et al., 1994).

Another possible reason related to the lack of asymmetries in the aMCI group, concerns the walking setting in our study. In most dual-tasks studies, participants are required to walk for shorter distances and in a linear fashion on a walkway. In our paradigm, subjects walked straight as well as negotiate the turns to follow the circuit within the walking area, which requires adjusting their walking accordingly. Memory and executive functions are necessary to maintain a safe gait, and deficits in these cognitive domains affect the ability to estimate hazards in balance and navigation (Montero-Odasso et al., 2017) such as the turns in the Optogait field. In a recent study, by Pieruccini-Faria et al. (2019) it has been demonstrated that executive functions have a mediating role in abnormal gait control and gait adjustments, meaning by this that persons with executive impairments cannot judge appropriately environmental hazards. Consequently, the walking design of the present study could have contributed to the prioritization of walking in aMCI individuals as turning poses additional challenges to walking (Sunderaraman et al., 2019).

In line with the previous argument, the counterclockwise direction adopted in our study may potentially have an impact on the results. To our knowledge, no study has been conducted to compare asymmetric effects of walking directions on spatio-temporal gait data and this yields for any type of population. However, a study by Caballero et al. (2019), showed no significant differences on walking kinematic variability between clockwise or counterclockwise walking directions. Thus, because right-handers tend to prefer a counterclockwise walking direction (Mohr et al., 2003), and walking turn preference has been reported to work as a stabilizing factor in walking (Lenoir et al., 2006) we believe that the adopted walking direction should not have a substantial effect on our results, at least on the cognitively healthy controls. Notwithstanding, data from our laboratory (Rodríguez-Aranda et al., 2018) and other researchers (Liu et al., 2018) indicate that right-handed MCI individuals undergo abnormal lateralized abilities that might cause alterations on walking preferences. Thus, future studies are encouraged to apply other walking alternatives to evaluate whether the present findings rely on the sole use of DL, independent of walking environment, or whether gait alterations due to DL are tightly related to the experimental situation.

All in all and based on the findings the most parsimonious interpretation is that our data point to a combination of auditory and attentional constraints that impeded good task-execution in aMCI individuals and hence, a lack of asymmetries. For this reason, we wish to deepen into the interplay of hearing, cognition and walking among aMCIs and healthy elders.

### Interplay of Hearing Loss, Attentional Abilities and Gait Perturbations in Normal Aging and aMCI

Results of the present study suggest that different levels of hearing loss and attentional decline in two groups of older adults interact differently during execution of dichotic listening while walking. The appearance of asymmetric effects on step length variability seems to be a perturbation related to normal aging, while the lack of asymmetries but exaggerated variability increments on gait needs to be regarded as pathological and proper to aMCI. These outcomes are of interest, and they contribute to better understanding the interplay of cognitive and sensory-motor changes in the aging continuum.

There are scarce empirical data about how concomitant disabilities such as hearing decline and attentional impairments affect functional aspects of older persons, such as gait. Much information exists coupling peripheral hearing loss with central auditory dysfunction, and risk factor for dementia (e.g., Thomson et al., 2017). Also, several cross-sectional and longitudinal investigations have reported a link between hearing loss, cognitive decline and frailty in older populations including community dwelling elders (Kamil et al., 2016), as well as those suffering of MCI and Alzheimer’s disease (Rahman et al., 2011; Panza et al., 2015; Wayne and Johnsrude, 2015). It must be highlighted that the link between hearing loss and cognitive decline in aging is not a new one (e.g., Lin et al., 2013). In contrast, the suggestion that these ailments are tightly related to frailty, and specifically to its operationalization based on gait impairments is a more recent observation (Ayers and Verghese, 2019; Panza et al., 2019; Cheng et al., 2021). Because, we are still far from understanding the real nature of these associations, we believe that the present study is a step forward to unveil how concrete cognitive constraints, such as attentional control dependent on hearing acuity affects gait in aMCI. In addition, our results have clinical implications since we have focused on aMCI, which is the MCI subtype most susceptible to progress into Alzheimer’s dementia. Since MCI subtypes are proposed to differ in neuropathology (see Doi et al., 2017), gait outcomes in dual-tasking are expected to vary accordingly. Though, so far, few dual-task studies have been conducted as an attempt to distinguish between non-amnestic and amnestic MCI (for review, see Doi et al., 2014; Montero-Odasso et al., 2014; Tseng et al., 2014; Bishnoi and Hernandez, 2020). The present findings suggest that in order to properly establish differential profiles based on MCI subtypes, cognitive and sensory declines need to be integrated.

### Limitations and Strengths

There are some limitations of this investigation that should be acknowledged. The lack of DL as single-task can be regarded as a weakness of our study. Many dual-tasks paradigms assess the motor and cognitive tests as both single and during dual-tasking. However, since we wished to evaluate the effects of the experimental situation without previous exposure to DL, we intentionally did not assess the cognitive test as single task. A similar approach has been adopted in several studies that equally have only assessed single-task performances in cognition (Al-Yahya et al., 2011). In the current study, the rationale of avoiding single-execution of DL was important to appraise the effects of this over-ground dual-task paradigm as a novel situation and as a more ecological approach where participants were naïve to the cognitive task. However, the interest of applying DL as single test among aMCI participants is evident and future research should include DL as a single-task (both the Bergen DL test and other variants) to deepen into the executive abilities of aMCI as well as to assess the effects of previous exposure of DL in the dual-task paradigm.

Another limitation is that we have not explored whether the walking direction (i.e., counterclockwise) and/or settings (i.e., walking in circles) may impact the results. Future investigations are encouraged to address these issues by conducting the present paradigm in straight walking environments and by comparing outcomes from different walking directions. Also, it is important to acknowledge that while our group of aMCI is well defined and the amnestic subtype is the most prone to convert into Alzheimer’s dementia (Ward et al., 2013), not everyone with such a diagnosis develop dementia (Langa and Levine, 2014). In fact, the certainty of the diagnosis can only be achieved after a follow-up assessment (Sun et al., 2019). This means that only through a longitudinal evaluation we would be able to assert whether the present paradigm can be used in the early detection of AD.

Despite the limitations of the present study, we wish to highlight some important strengths. In addition to the application of an ecologically valid paradigm, we regard the selection of the patient group as important. The aMCI participants recruited in our study were referred from the University Hospital of North Norway with a clinical diagnosis of MCI. Thereafter, these participants underwent a thorough neuropsychological assessment, which enabled correct classification into MCI subtypes. Compared to many aging studies dealing with a wide category of MCI, who are recruited from the community and are often categorized in MCI upon single measures of cognitive status (e.g., MMSE score), our criteria for aMCI inclusion provides clinical trustworthiness to our findings. We used several measures within each cognitive domain to determine not only subtype of MCI but also to ensure normal cognitive status of controls. Many studies apply too few measures representing different cognitive domains, which prove not to be sufficient (Clark et al., 2013). By having the certitude that the MCI group in this current study is properly classified as aMCI, we also assert that this sample indeed displays mixed difficulties of memory and executive dysfunctions. Rabi et al. (2020) suggested that individuals properly categorized as aMCI from clinical samples, not only show higher conversion to Alzheimer’s Disease but also perform significantly worse on measures of executive functions than community-based samples. This in turn allows us to claim that the difficulties to perform DL task by the aMCI group are strongly related to executive impairments and higher levels of hearing loss.

### Clinical Implications and Future Directions

Challenging everyday actions such as dual-tasking depend heavily on cognitive resources but also on adequate hearing and free walking. The present approach reveals the importance to assess multiple bodily and cognitive changes affecting older adults that are in need of preserving their autonomy as long as possible. Applying DL with gait assessment may provide a cost-efficient and sensitive measure to detect gait difficulties, cognitive dysfunction, and auditory difficulties in older adults with a probable risk of developing dementia. Since older adults with hearing loss are at greater risk of falls, audiological assessment in addition to thorough cognitive evaluation and gait analysis may be important in providing a holistic approach to aid activities of daily living in older adults with MCI. The association between cognition, hearing loss and gait disturbances provides an interdisciplinary approach in assessment and shows that a targeted audiological rehabilitation could be used to complement physical and cognitive rehabilitation in older adults.

Furthermore, we consider that the clinical application of the present paradigm has a great potential on the differential diagnosis of various MCI subtypes. For instance, results of the present study can be compared to the rest of the traditional MCI subgroups. Though, because our paradigm tightly involves a motor element (walking), hearing ability and their interplay to lateralized attentional/executive capacities, other MCI subtypes more prone to present impairments in these areas represent a fruitful venue of exploration. The recent criteria proposed for prodromal Lewy-Body Dementia (LBD) and Parkinson’s Disease Dementia (PDD) (McKeith et al., 2020; Pieruccini-Faria et al., 2021) offers good examples. It has been reported that auditory hallucinations are an important characteristic for LBD and PDD (Eversfield and Orton, 2019) and in turn, hallucinations have been related to greater hearing loss, mainly in PDD (Lai et al., 2014). In addition, it is suggested that MCI for LBD and PDD characterizes by important executive impairment which has been successfully evaluated with the Stroop test (Belghali et al., 2017). Thus, application of the present paradigm with dichotic listening offers a good alternative that relies on an ecologically valid environment. In sum, it is appealing to consider in future research the use of dichotic listening while walking in the differential diagnosis of prodromal LBD and PDD.

## Conclusion

Results of the present study demonstrate that the interplay between cognitive status, hearing loss and gait perturbations differs between cognitively healthy older adults and individuals with aMCI. Asymmetric effects on step length variability were evident in controls who were able to perform DL task appropriately. In contrast, symmetric gait variability increased overly in aMCI participants due to lack of cognitive and auditory abilities that enabled them to execute the DL test. Thus, the association between hearing, cognition and gait in older populations is undisputable, but based on our findings the interactive mechanisms are not so easy to seize. Outcomes may depend upon degree of impairment and task difficulty. In addition, other factors such as task prioritization, novelty in the walking environment and practice may have a further impact in the results. Future studies should further investigate the importance of these aspects in different MCI subtypes.

Application of the present dual-task paradigm with aMCI individuals stresses the importance of considering sensory loss when assessing the mechanisms behind dual-task decrements in older adults with cognitive impairment. From a clinical perspective, it is crucial to understand the moderating role of hearing loss in cognition and functional abilities, especially related to how these deteriorations enhance the risk of dementia development. Therefore, we consider that the present paradigm is a suitable alternative to better understanding of the sensory-motor-cognition triad of hearing loss, gait perturbations and executive impairments in MCI.

As exposed by authorities in the field, there is a need to improve the methods used to understand the cognition-gait association link in specific populations of older adults (Montero-Odasso et al., 2019). Currently, the cognitive tasks suggested for dual-task paradigms rely on complex and intertwined cognitive abilities with no predominant involvement of a specific sensorial modality. Probably therefore they affect gait at a rather general level, perturbing many spatiotemporal parameters. Thus, we believe that the present findings are a step forward to improve an understanding of how specific attentional constraints in the auditory modality affects concrete gait characteristics. It is still early to declare whether our paradigm is a suitable assessment method for the detection of aMCI as we have to assert adequate difficulty level of DL and the possible differential strength of this method for different MCI subtypes at the long term. Nevertheless, application of dichotic listening on dual-task paradigms provides a promising multicomponent assessment tool for the early detection of cognitive impairment and future studies should account for other decrements in sensory functions such as visual acuity or balance.

## Data Availability Statement

The datasets presented in this article are not readily available because this study is part of an ongoing umbrella project and data will be available upon request to the PI at the end of data collection. Requests to access the datasets should be directed to CR-A, claudia.rodriguez-aranda@uit.no.

## Ethics Statement

The studies involving human participants were reviewed and approved by Regional Committee for Medical and Health Research Ethics—REK (2009/1427). The patients/participants provided their written informed consent to participate in this study.

## Author Contributions

CR-A and MG contributed with the conception and design of the study, performed the statistical analyses, and wrote the draft. MG and OV recruited participants and collected the data. KW helped with the overall logistics and revised intellectual content. All authors contributed to manuscript revision, read, and approved the submitted version.

## Conflict of Interest

The authors declare that the research was conducted in the absence of any commercial or financial relationships that could be construed as a potential conflict of interest.

## Publisher’s Note

All claims expressed in this article are solely those of the authors and do not necessarily represent those of their affiliated organizations, or those of the publisher, the editors and the reviewers. Any product that may be evaluated in this article, or claim that may be made by its manufacturer, is not guaranteed or endorsed by the publisher.
