# Supplementary material for: Assessing a Sensory-Motor-Cognition Triad in Amnestic Mild Cognitive Impairment With Dichotic Listening While Walking: A Dual-Task Paradigm
Source: Front Aging Neurosci. 2021 Nov 12;13:718900. doi: 10.3389/fnagi.2021.718900 (PMC8633416; doi:10.3389/fnagi.2021.718900)
Supplement: Supplementary file 1 [file Table_1.docx]

TABLE 1 SUPPLEMENTARY MATERIAL

Results for bilateral gait parameters by group

**CONDITION**

**Baseline** **Non-Forced** **Forced-Right** **Forced-Left**

Controls aMCI Controls aMCI Controls aMCI Controls aMCI RMANOVA, *p, (η²_ₚ_)* ANCOVA*, p, (η²_ₚ_)*

*M (SD) M (SD) M (SD) M (SD) M (SD) M (SD) M(SD) M(SD)* Cond./Interac./Group Interac./Group/PTA

**Mean**

Step length 64.0 (8.9) 55.5 (8.9) 60.6 (9.6) 51.8 (9.3) 59.1 (9.5) 50.9 (8.8) 59.1 (9.6) 50.4 (9.1)  **0.001 (0.5)**/ NS**/0.001 (0.9)**  NS**/0.001 (0.3)**/**0**.**003 (0.1)**

Gait speed 1.1 (0.2) 0.8 (2.2) 1.0 (0.2) 0.8 (0.3) 0.9 (0.3) 0.7 (0.2) 0.9 (0.3) 0.7 (0.3) **0.001 (0.5)/**NS/**0.001 (0.2)**  NS/**0.001 (0.3)/0.003 (0.1)**

Step width 9.3 (3.4) 13.2 (3.1) 9.5 (2.8) 13.1 (3.2) 10.0 (4.1) 13.1 (3.6) 10.3 (3.7) 13.3 (3.7) NS / NS/ **0.001 (0.2)** NS/**0.001 (0.1) /0.001 (0.1)**

**CoV (%)**

Step length 7.5 (6.1) 14.2 (6.8) 8.3 (5.9) 15.7 (7.1) 9.6 (7.0) 15.7 (7.2) 8.9 (6.5) 15.8 (7.1) **0.001(0.0)**/NS /**0.001(0.2)** NS**/0.001(0.1) /0.001 (0.2)**

Gait speed 8.5 (9.3) 20.6 (11.6) 9.4 (8.7) 21.6 (10.7) 12.8 (15.5) 25.8 (29.7) 11.9 (14.5) 26.6 (35.7) NS / NS / **0.001(0.2)** NS**/0.001(0.1)/0.027(0.1)**

Step width 87.0 (46.9) 93.6 (37.6) 81.9 (30.9) 85.2 (30.1) 87.3 (29.7) 87.9 (29.5) 87.1 (34.7) 85.2 (27.9) NS / NS / NS  NS/ NS / NS

*Note*. RMANOVA and ANCOVA with Bonferroni correction for multiple comparisons. Units for Step length, Step width and Stride length = cm.; units for Gait speed = m/sec. Abbreviations: *M* = mean; *SD* = standard deviation; RMANOVA = repeated measures analysis of variance; ANCOVA = Analysis of covariance; CoV = Coefficient of Variation; Interac. = Interactions; PTA = Best Pure Tone Audiometry values; NS = Non Significant; CoV = Calculated with the formula: [mean/ SD] x 100%
